# Grazing-incidence diffraction reveals cellulose and pectin organization in hydrated plant primary cell wall

**DOI:** 10.1038/s41598-023-32505-8

**Published:** 2023-04-03

**Authors:** Joshua T. Del Mundo, Sintu Rongpipi, Hui Yang, Dan Ye, Sarah N. Kiemle, Stephanie L. Moffitt, Charles L. Troxel, Michael F. Toney, Chenhui Zhu, James D. Kubicki, Daniel J. Cosgrove, Esther W. Gomez, Enrique D. Gomez

**Affiliations:** 1 Department of Chemical Engineering, The Pennsylvania State University, University Park, PA 16802 USA; 2 Department of Biology, The Pennsylvania State University, University Park, PA 16802 USA; 3 SLAC National Accelerator Laboratory, Menlo Park, CA 94025 USA; 4 Department of Chemical and Biological Engineering and the Renewable and Sustainable Energy Institute, University of Colorado Boulder, Boulder, CO 80309 USA; 5 Advanced Light Source, Lawrence Berkeley National Laboratory, 1 Cyclotron Road, Berkeley, CA 94720 USA; 6 Department of Earth, Environmental and Resource Sciences, University of Texas at El Paso, El Paso, TX 79968 USA; 7 Department of Biomedical Engineering, The Pennsylvania State University, University Park, PA 16802 USA; 8 Department of Materials Science and Engineering, The Pennsylvania State University, University Park, PA 16802 USA; 9 Materials Research Institute, The Pennsylvania State University, University Park, PA 16802 USA

**Keywords:** Plant sciences, Soft materials

## Abstract

The primary cell wall is highly hydrated in its native state, yet many structural studies have been conducted on dried samples. Here, we use grazing-incidence wide-angle X-ray scattering (GIWAXS) with a humidity chamber, which enhances scattering and the signal-to-noise ratio while keeping outer onion epidermal peels hydrated, to examine cell wall properties. GIWAXS of hydrated and dried onion reveals that the cellulose ($110/1\overline{1}0$) lattice spacing decreases slightly upon drying, while the (200) lattice parameters are unchanged. Additionally, the ($110/1\overline{1}0$) diffraction intensity increases relative to (200). Density functional theory models of hydrated and dry cellulose microfibrils corroborate changes in crystalline properties upon drying. GIWAXS also reveals a peak that we attribute to pectin chain aggregation. We speculate that dehydration perturbs the hydrogen bonding network within cellulose crystals and collapses the pectin network without affecting the lateral distribution of pectin chain aggregates.

## Introduction

In plants, the primary cell wall provides structural integrity, imparts tissue flexibility, and mediates growth in response to cell turgor pressure. The components of primary cell walls include crystalline cellulose microfibrils (CMFs) embedded in a matrix of pectin, xyloglucan, and endogenous proteins. The structural properties of these components and the interactions between them govern the mechanical properties of the primary cell wall as a whole^1,2^. Primary cell wall is also a material source for a variety of products. Pectin is used as an additive in food industries, and in pharmaceutical and biomedical applications in recent years^3^. Cellulose is the major feedstock for textiles, paper, lumber, and biofuels. Nevertheless, the degradation of cellulose for use in these industries has been a historical challenge^4^. Detailed material characterization of primary cell walls is needed to develop more efficient pathways for conversion of its components into commodity chemicals, fuels, and bio-inspired materials.

X-ray diffraction (XRD), often termed wide-angle X-ray scattering (WAXS) when in a transmission geometry, has been used to probe crystalline properties of cellulose, such as lattice spacing, crystallite size, and crystallinity^5–7^. The structure of native pectin on the other hand is less established. A previous study suggested that pectin in dried mung bean primary cell walls contributes to a broad amorphous peak in WAXS data that corresponds to a circa 4.5 Å spacing^5^. Nevertheless, XRD of pectin varies greatly based on source and extraction method. While a spacing of about 4.5 Å in pectin has been reported in many cases^8–10^, wider spacings ranging from 6.5 to 8.0 Å have also been observed^8–12^. Overall, the structural properties of hydrated pectin in the native state remain unclear.

The high level of hydration of primary cell wall leads to challenges in characterizing crystalline properties. X-ray scattering studies often examine dried samples, but this could be an inaccurate representation of the native state. Upon drying, plant tissues decrease in size and porosity^13,14^. Primary cell walls undergo an increase in modulus and exhibit collapse of the pectin matrix^15,16^. As such, shrinking or denaturation that may result from these changes can lead to artefacts in structural studies^17^. Large amounts of water in a sample, however, will produce a strong peak at around 2.0 Å^−1^ that often interferes with the scattering from cellulose. In one example of a powder XRD study, the water peak overwhelmed the cellulose peaks in hydrated *Acetobacter* cellulose^18^.

Raman spectroscopy of never-dried wood cellulose suggests the possibility that water binds to internal cellulose chains in “water accessible” regions as well as at the CMF surface^19^. To further expand this hypothesis, and how that would in turn affect diffraction, we use density functional theory (DFT) simulations of 18-chain microfibrils^20,21^. We consider the possibility that water interacts with both the surface and internal chains of the cellulose crystal. The (200) lattice spacing, the distance between cellulose sheets, is around 4 Å. This is large enough to contain a water molecule with a 2.8 Å diameter^22^. Our DFT models thus simulate the effects of dehydration on CMFs with and without water molecules in between cellulose sheets.

To overcome the experimental challenge of probing a highly hydrated system, we employ grazing-incidence wide-angle X-ray scattering (GIWAXS). Grazing-incidence scattering near the critical angle for total external reflection provides a large sample probing area and consequently a high signal-to-noise ratio^23^. Additionally, grazing-incidence has the capability to decouple scattering features out-of-plane from in-plane with respect to the sample. Previous GIWAXS work on primary cell walls, however, was conducted on dried samples^24^.

Here, we use GIWAXS to examine the hydrated and dehydrated states of onion outer epidermal peel, a model system for primary cell walls. Peeling the outer epidermal layer from an onion scale ruptures the outermost layer of cells, exposing the newly deposited primary cell wall surface, and makes the sample amenable to various characterization techniques, such as microscopy and scattering^25^. An excess amount of water, however, will dominate scattering data. To mitigate this issue, we employ a humidity chamber to preserve the hydration of the onion peel without the need for immersion. We find changes in cellulose lattice spacing and relative intensities of ($110/1\overline{1}0$) and (200) reflections, which we speculate occur from dehydration. These results are qualitatively consistent with our DFT models. A scattering feature from pectin does not diminish upon drying, suggesting a lateral aggregation that persists upon collapse of the pectin network from dehydration.

## Materials and methods

### DFT calculations

Recent evidence suggests that the shape of primary CMFs in plant cell walls is a 6-layer CMF in an arrangement of 234432 glucan chains where each integer represents the number of chains in a given layer^20^. Here, for direct comparison against experimental results, four 234432 models with different water contents were obtained via energy minimization conducted with Vienna Ab-initio Simulation Package (VASP)^20,26–30^. Model 1 is comprised of a CMF without any H_2_O molecules (72C_6_O_5_H_10_), Model 2 of a CMF with a monolayer of H_2_O molecules [72C_6_O_5_H_10_ + 102H_2_O (outside)], Model 3 of a CMF with H_2_O molecules inside the cellulose microfibril [72C_6_O_5_H_10_ + 36H_2_O (inside)], and Model 4 of a CMF with H_2_O molecules inside the cellulose microfibril plus a monolayer of H_2_O molecules [72C_6_O_5_H_10_ + 36H_2_O (inside) + 108H_2_O (outside)].

Initial atomic structures of CMF Model 1 were based upon previously reported cellulose structure^31^. The unit cell of cellulose was doubled to include 4 glucan units along the c-dimension, and 18 polymer CMFs were constructed in Materials Studio 2016. Initial atomic structures of CMF with a monolayer of H_2_O molecules in Model 2 were based upon classical molecular dynamics (MD) simulations^20,32^. From the last step of the classical MD simulation, the 18 -chain fibril plus all H_2_O molecules within 3 Å of the fibril (i.e., a “monolayer” of hydration) were extracted for DFT energy minimizations. Initial atomic structures of Model 3 and 4 were generated by manually placing H_2_O molecules, the number of which was arbitrarily selected that could fit within the CMF in Model 1 using the Visualizer module of Materials Studio. The interior H_2_O molecules then were relaxed through Geometry Optimization using the COMPASSII force field in Materials Studio while the atoms of the CMF were constrained to the positions originally derived from DFT-D3 calculations. D3 denotes the D3 dispersion correction of Grimme et al. using the VASP code^33^. The DFT-D3 dispersion energy correction considers not only all pairs of atoms but also triplets of atoms to account for three-body effects. It is an add-on term that does not directly alter the wave function. The model structures were converted into VASP input files (POSCAR) via a Perl script written by Andrei V. Bandura (Saint Petersburg State University, Russia) within a simulation cell of 40 × 40 × 40 Å^3^. All atomic positions were relaxed during energy minimizations with periodic DFT-D3 calculations^34,35^ with PBE0 pseudopotentials, ENCUT = 500 eV, D3 dispersion correction of Grimme et al. using the VASP code^26,27,29,33,36^. After convergence of the chain lengths, the lattice parameters were then held constant in order to prevent artificial aggregation of the CMFs with their periodic images (i.e., contraction of the a and b cell dimensions until the CMF would sense images of itself).

To compare against experimental results, scattering curves of CMF Models 1–4 in aqueous solution were calculated based on explicit-solvent all-atom MD simulations using WAXSiS^37,38^. The 18-chain CMF structures were obtained based on the energy minimizations conducted with VASP. Each chain contained 16 glucose units. Each CMF structure was solvated in a box of TIP3P water so that the CMF was surrounded in all directions by 20 Å. Short (50 ps) MD simulations were performed using AMBER^39^ and the Glycam06 carbohydrate force field at 300 K, at 1 bar, in the NPT ensemble, with 2 fs time step, 10 Å non-bonded interaction cutoff, and SHAKE-constrained hydrogen bonds. The positions of the CMF were constrained using a force constant of 250 kcal/mol/Å^2^. Simulation frames were written every 0.5 ps so that the solvent configurations were uncorrelated^38^. From the MD trajectories, the theoretical scattering curves were then calculated using the WAXSiS webserver. The scattering from cellulose crystals *I*(*q*) is calculated by subtracting the scattering from the surrounding TIP3P water *I*_*buffer*_(*q*) using *I*(*q*) = *I*_*sample*_(*q*) − (1 − *v*) *I*_*buffer*_(*q*), where *v* is the volume fraction of the solute and *I*_*sample*_ is the scattering from the CMF and solvent^38^.

### Sample preparation

White onions (*Allium cepa*, cv. Cometa, ca. 7 cm in diameter) were purchased from a local grocery store. Epidermal peels from the abaxial side of the 5^th^ scale of the onion (counting from the outside) were removed using previously described methods^24,25^. The peels were extracted in 20 mM HEPES buffer at pH 6.8 containing 0.1% Tween-20 for 1 h to remove cellular debris. Samples were then washed six times in deionized water and washed once in 2 mM sodium azide to prevent bacterial growth. For hydrated GIWAXS samples, the peels were mounted onto cleaned silicon wafers, cuticle-side down, and stored in between glass slides surrounded by moist tissue paper to preserve hydration.

For the identification of amorphous cell wall components, GIWAXS of enzymatically treated peel was conducted. For the pectate lyase treatment, samples were washed with 0.1% Tween 20 in 20 mM MES at pH 6.8 for approximately 12 h and then incubated in 20 μg/ml pectate lyase (Megazyme E-PLYCJ, *Cellvibrio japonicus*) in 20 mM TRIS with 5 mM CaCl_2_ at pH 9.5 and 37 °C for approximately 12 h while on an orbital shaker at 50 rpm. The enzyme used in this experiment had a pH optimum of 10.0. Potassium hydroxide was used to adjust the pectate lyase buffer to pH 10 for optimum activity of the enzyme. After pectate lyase treatment, the samples were rinsed in DI water six times, mounted onto silicon wafers, and air-dried. Driselase treatment for GIWAXS characterization was performed as previously described^24^.

### GIWAXS and rocking scan measurements

Hydration experiments were conducted at beamline 11–3 of the Stanford Synchrotron Radiation Lightsource. The humidity chamber is composed of a water reservoir and sponge placed inside the sample chamber. Samples were equilibrated in the chamber for at least 20 min, after which time the relative humidity (RH) reaches about 65% (Supplementary Fig. S1). Gravimetric measurements of water loss in air indicate that onion peels are 91% water, and that samples remain translucent until 80% water content (Supplementary Fig. S2). Thus, although no water loss is apparent in GIWAXS samples stored within the humidity chamber, we can also confirm that samples used for scattering measurements contain more than 80% water, or that the water loss is less than 12%. GIWAXS measurements were performed with a 12.7 keV X-ray beam at an incident angle of 0.12°. Rocking scan measurements were collected at 5.07 ± 0.73°, near the ($110/1\overline{1}0$) reflection. Scattering was collected with a Raxyonics 225 detector. Hydrated samples were measured in the humid air environment. Afterwards, the samples were air-dried for 12 h and measured again in a helium environment. Enzymatically digested samples were measured at the Advanced Light Source beamline 7.3.3. GIWAXS measurements were performed with a 10.0 keV X-ray beam at an incident angle of 0.15°. Scattering was collected with a Pilatus 2 M detector.

GIWAXS and rocking scan images were analyzed using WxDiff^40^ and Xi-Cam^41^ as previously described^24^. We denote χ as the azimuthal angle around the beam center, where χ = 0° is defined to be vertical direction above the beam center. Out-of-plane profiles were obtained by integrating sectors at χ = − 17° to 17°. A linear baseline background was subtracted from the out-of-plane profile sector cuts. In-plane profiles were obtained by integrating sectors at χ = 56° to 90°. Azimuthal intensity profiles of the ($110/1\overline{1}0$) reflection in GIWAXS and rocking scan data were integrated from q = 1.0 to 1.3 Å^−1^. Azimuthal intensity profiles integrated from q = 0.5 to 0.6 Å^−1^ were taken as backgrounds for both GIWAXS and rocking scan data and were subtracted from the ($110/1\overline{1}0$) reflections. GIWAXS and rocking scan azimuthal intensity profiles were reduced and stitched into complete ($110/1\overline{1}0$) χ pole figures as previously described^24,42^.

### Thermogravimetric analysis

Thermogravimetric analysis (TGA) was conducted using a Discovery Series TGA Q5500 coupled with a Discovery mass spectrometer (TA Instruments) in an argon environment, from 25 to 250 °C at a rate of 10 °C/min. Onion epidermal peels used in TGA were separate from those used in X-ray measurements. 10 mg of hydrated onion peel and 1.5 mg of air-dried onion peel were measured. Dried samples were exposed to the furnace isothermally at 25 °C for 30 min before heating to remove absorbed water. Water content was determined by calculating the weight percent difference between 25 and 150 °C. Data are reported as mean ± standard deviation (n = 3). Statistical analysis used a Student’s t-test.

## Results

### DFT models

**Figure 1:**
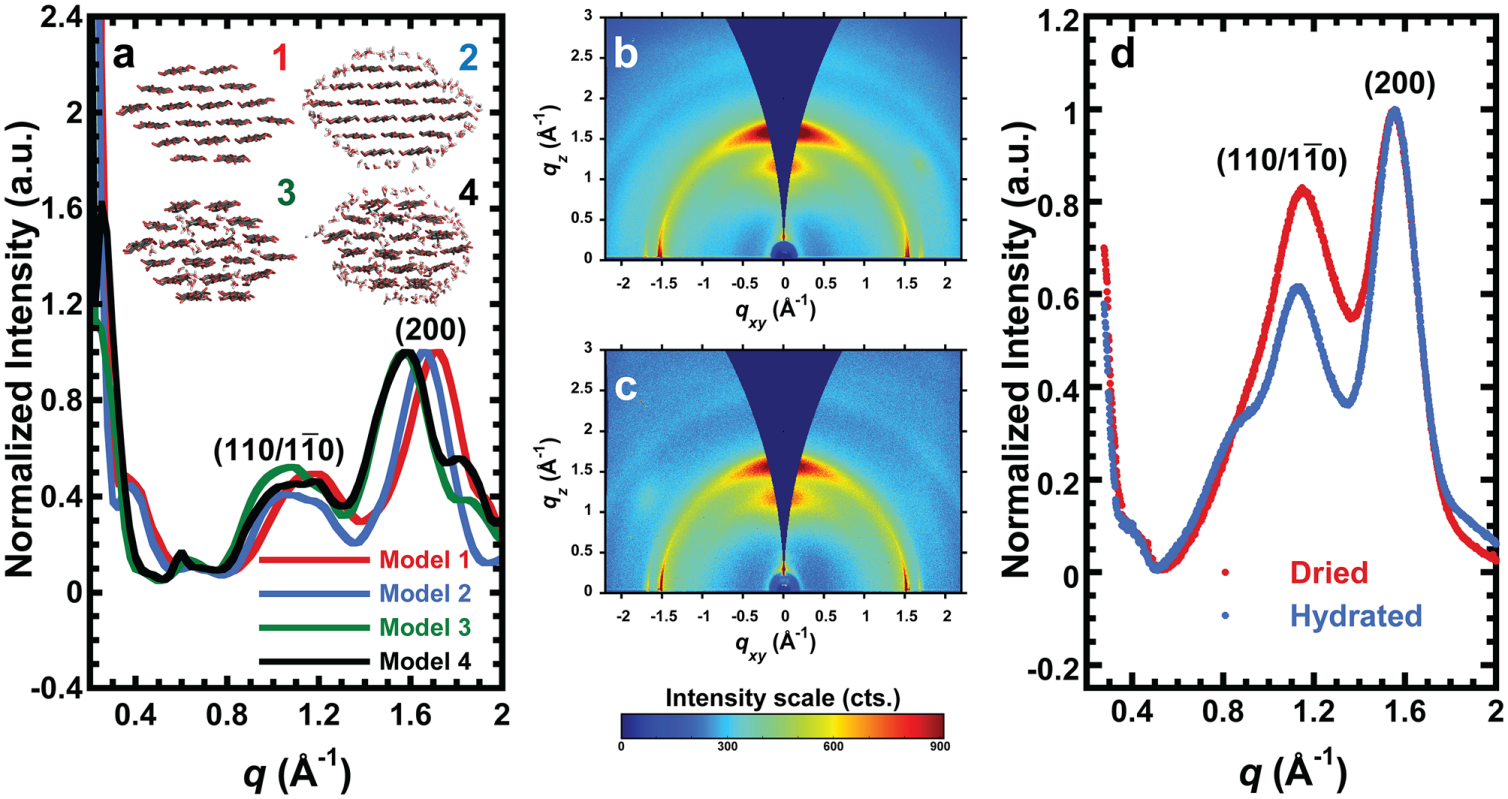
Theoretical and experimental X-ray scattering data. (**a**) Predicted scattering curves of CMF models normalized to the (200) maximum peak intensity. Inset: Potential crystal habits of cellulose microfibrils with (1) no H_2_O molecules, (2) monolayer of H_2_O molecules on the outside of the cellulose crystal, (3) H_2_O molecules inside the cellulose microfibril, and (4) H_2_O molecules inside the cellulose microfibril and a monolayer of H_2_O molecules on the outside surface. (**b**,**c**) GIWAXS data for onion epidermal wall in (**b**) hydrated and (**c**) dried states. *q*_*z*_ and *q*_*xy*_ denote out-of-plane and in-plane scattering vectors, respectively. (**d**) GIWAXS out-of-plane profiles of hydrated (blue) and dried (red) onion epidermal wall. A linear background was subtracted and data are normalized to the (200) peak intensity for comparison to (**a**). Profiles are averaged over three replicates.

Four DFT models (Fig. 1a, inset) explore the effect of water on the structure of an 18-chain elementary CMF. Water monolayers simulate the effects of bulk water around a cellulose Iβ crystal. Cellulose Iβ is the most dominant allomorph in higher plants, although CMFs usually consist of a mixture of Iβ and Iα^43,44^. We consider a dry cellulose crystal (Model 1) and a crystal with a water monolayer on the outside of the crystal (Model 2). We also consider the possibility of water inside the crystal between layers of glucan chains (Model 3), and water inside and as a single layer outside the crystal (Model 4). The removal of surface-bound water and embedded water is highly unlikely from air drying^45^. We compare Model 1 (dry) and Model 2 (hydrated) when we assume there is no water inside crystals, and Model 3 (dry) and Model 4 (hydrated) when we consider the possibility of water molecules within crystals.

Simulated diffraction patterns are shown in Fig. 1a as scattering intensities versus scattering vector (scattering vector *q* = 4πsin*θ*/*λ*, where *θ* is one half of the scattering angle, and *λ* is the X-ray wavelength). Diffraction peaks that correspond to the ($110/1\overline{1}0$) and (200) lattice spacings are near 1.1 and 1.5 Å^−1^_,_ respectively (Fig. 1a), which are roughly consistent with published lattice parameters of cellulose Iβ^31^. The ($110/1\overline{1}0$) region appears to have multiple peaks, possibly representing the individual (110) and ($1\overline{1}0$) reflections. The (200) lattice spacing varies within the models (Supplementary Table S1). Model 1 has smaller ($110/1\overline{1}0$) and (200) lattice spacings than Model 2, as indicated by shifts in peak positions. The peak positions between Models 3 and 4 are similar, although shifts corresponding to the ($110/1\overline{1}0$) reflection are more challenging to interpret. Thus, when water is not present within the crystal, hydrated models exhibit larger lattice constants, while when water is included within microfibrils, lattice spacings stay relatively constant regardless of the presence of water outside crystals. In addition, our models with a surface water monolayers have a higher ($110/1\overline{1}0$) intensity relative to (200) than the models without external water.

### GIWAXS and rocking scans

Previous GIWAXS data of dried onion epidermal wall reveals that cellulose crystal planes are textured out-of-plane and that scattering from cellulose can be decoupled from that of cuticular wax, which has features that are textured in-plane with respect to the cell wall surface^24^. The decoupling of these signals allows GIWAXS to differentiate scattering from cellulose and cuticle without the need for cuticle removal treatments. Building on this work, GIWAXS of hydrated and dried onion epidermal cell wall are shown in Fig. 1b,c. In both the hydrated and dried samples, ($110/1\overline{1}0$) and (200) reflections that are characteristic of cellulose Iβ are broadly distributed about the *q*_*z*_ axis, indicating out-of-plane texturing with multiple populations, as previously described^24^. The features along the *q*_*xy*_ axis are textured in-plane and are attributed to cuticle wax crystal planes.

Reduced 1D scattering profiles of the out-of-plane GIWAXS data shown in Fig. 1d reveal ($110/1\overline{1}0$) and (200) peak positions near 1.1 and 1.5 Å^−1^_,_ respectively, which are consistent with the cellulose Iβ structure^31,46^. The (110) and ($1\overline{1}0$) reflections appear as a single peak, which has been observed in WAXS of primary cell walls. The merging of the two reflections is attributed to small crystallite size and disorder in cellulose Iβ^5,6,24,47^. We interpret the position of the combined ($110/1\overline{1}0$) peak as the average between the (110) and ($1\overline{1}0$) lattice spacings. A feature at around 0.4 Å^–1^ in the GIWAXS data is also present in DFT models 1 and 2. Some features are present in the theoretical scattering curves but not in the experimental data, at around 1.9 Å^−1^ in models 3 and 4, and at around 0.6 Å^−1^ for all models (Fig. 1a).

**Figure 2:**
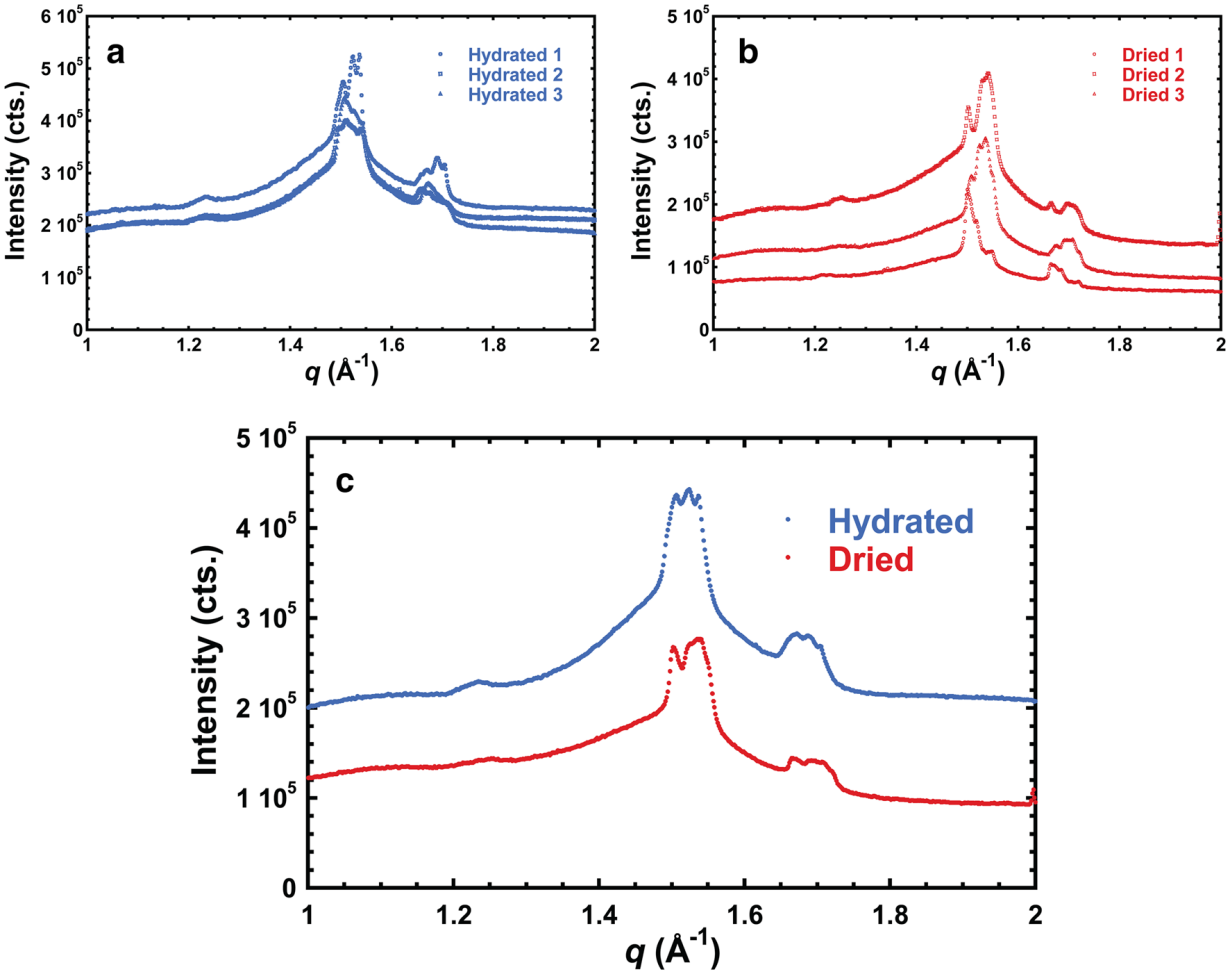
In-plane GIWAXS profiles of (**a**) three hydrated onion epidermal walls, and of (**b**) three dried onion outer epidermal walls. (**c**) Comparison of averaged in-plane GIWAXS profiles of hydrated and dried samples shown in (**a**) and (**b**).

The in-plane GIWAXS profiles (Fig. 2) contain sharp features between 1.45 and 1.55 Å^−1^ and between 1.65 and 1.75 Å^−1^, which are attributed to cuticular wax crystals^24^. The peak positions vary between samples, resulting in multiple peaks visible in averaged profiles (Fig. 2c). The multiple peaks could arise from the presence of multiple orientations or populations (i.e., multiple allomorphs) of crystals in the cuticle.

**Figure 3:**
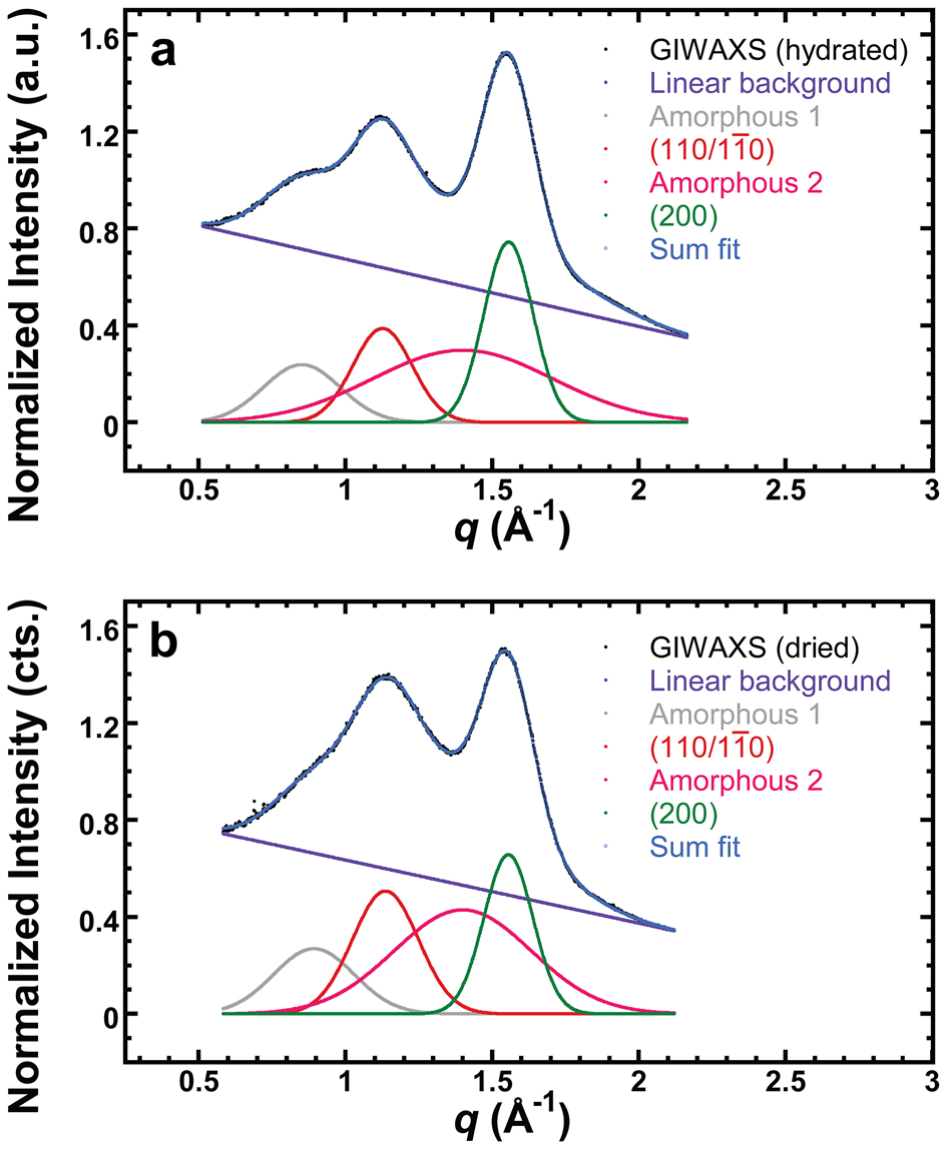
Examples of peak deconvolutions of the out-of-plane profiles from GIWAXS of (**a**) hydrated and (**b**) dried onion epidermal wall. GIWAXS data is shown in black (GIWAXS out-of-plane profile), and components that make up a fit to the data (Sum Fit) are also shown. Amorphous 1 and Amorphous 2 primarily arise from pectin and cuticle, respectively. The ($110/1\overline{1}0$) and (200) reflections are from crystalline cellulose for the Iβ allomorph.

To identify amorphous components, GIWAXS was performed on enzymatically-treated onion peels. Scattering from onion peels treated with pectate lyase, which removes pectin from the cell wall, leads to a decrease in the intensity of one of the broad peaks (at lower *q*, Amorphous 1 in Fig. 3 and Supplementary Fig. S3). We thus attribute this peak to scattering from pectins in the cell wall. A small decrease in the intensity of the higher *q* broad peak (Amorphous 2 in Fig. 3 and Supplementary Fig. S3) is also observed, although the peak appears dominated by another component. Scattering from cuticle was measured by removing all other cell wall components from onion epidermis using Driselase, which is an enzyme mixture that degrades cellulose, hemicellulose, and pectin. As shown in Supplementary Fig. S4, Driselase-treated samples show a broad out-of-plane reflection at *q* = 1.41 Å^−1^ that can be described as a Gaussian peak. Previous work attributes this feature to pectin^5^ as well as amorphous cellulose and hemicellulose^48^. Nevertheless, Supplementary Fig. S4 shows that cuticle is the most prominent component of this broad reflection (Amorphous 2 in Fig. 3).

We deconvoluted the out-of-plane GIWAXS profiles by fitting two broad Gaussian amorphous components, two Gaussian crystalline cellulose reflections from the ($110/1\overline{1}0$) and (200) planes, and a linear instrumental background extrapolated from high q (Fig. 3). The fit parameters are summarized in Supplementary Table S2. The low *q* broad peak corresponding to scattering from pectin (Amorphous 1) is at *q* = 0.85 Å^−1^ for hydrated and *q* = 0.89 Å^−1^ for dried samples (p < 0.01). A scattering contribution from cuticle (Amorphous 2) is fixed at *q* = 1.41 Å^−1^, although the peak width decreases from 0.77 Å^−1^ to 0.54 Å^−1^ upon drying. Furthermore, as shown in Table 1, the ($110/1\overline{1}0$) intensity increases and this reflection shifts to higher *q* upon drying, corresponding to a d-spacing change from 5.57 Å when hydrated to 5.52 Å (*d* = 2π/*q*) when dry. In contrast, the (200) peak position does not change between hydrated and dried samples.

**Table 1:** Cellulose lattice spacings of the ($110/1\overline{1}0$) and (200) planes and the ratio of the peak intensity at ($110/1\overline{1}0$) to that of (200) from the out-of-plane GIWAXS profiles of hydrated and dried onion epidermal wall. Values shown are the sample mean ± standard deviation, where n = 3. p-values obtained by comparing hydrated and dried using a paired student’s t-test. *p < 0.05 for comparison between hydrated and dried samples.

	d($110/1\overline{1}0$) (Å)	d(200) (Å)	I($110/1\overline{1}0$)/I(200)
Hydrated	5.57 ± 0.01*	4.03 ± 0.01	0.52 ± 0.02*
Dried	5.52 ± 0.01	4.02 ± 0.01	0.86 ± 0.04

The coherence length *L*, which is related to the crystal size, was calculated from the width of the scattering peaks using the Scherrer equation (L = Kλ/βcos*θ*), where *λ* is the X-ray wavelength in Å, β is the scattering angle full width at half maximum (FWHM) in radians after an instrumental broadening correction, *θ* is one half of the peak scattering angle, and *K* is a dimensionless shape factor taken to be 0.9^49^. In general, the peak width is affected by several factors, including the crystal size, paracrystallinity, and non-uniform strain^50,51^. The ($110/1\overline{1}0$) peak is composed of two reflections, such that any changes could be due to changes in position of one of the constituent peaks or broadening of either peak. Nevertheless, the width of the ($110/1\overline{1}0$) reflection increases from 0.240 to 0.277 Å^−1^ after drying (Supplementary Table S2). The peak width or coherence length of the (200) plane does not change significantly after drying (Supplementary Table S3).

The ($110/1\overline{1}0$) peak intensity relative to (200) is significantly higher in dried samples than in hydrated samples, as shown in Fig. 1d, where scattering intensities are scaled to the (200) peak. Table 1 compares the relative intensity of these two reflections for dry and hydrated samples after peak fitting.

The amorphous peak positions used in the fitting model of Fig. 3 are consistent with the scattering results from enzymatic digestion of onion cell wall. We also explored other choices of fitting parameters and amorphous backgrounds. Changing the position of Amorphous 2 does not affect the apparent decrease in the ($110/1\overline{1}0$) spacing with drying; for example, if Amorphous 2 is centered at q = 1.2 Å^−1^ instead of 1.41 Å^−1^, the contraction of ($110/1\overline{1}0$) is still present (Supplementary Fig. S5a,b, Supplementary Table S4). Introducing asymmetry to background peaks also does not appear to affect any trends. Applying an asymmetric peak function for Amorphous 1 changes the values for d-spacing and intensity ratio slightly, although all trends are preserved (Supplementary Fig. S5c,d, Supplementary Table S4). A fit that incorporates a smooth, broad asymmetric background also results in the same trends (Supplementary Fig. S5e,f, Supplementary Table S4). Thus, we present Fig. 3 as our best estimate of appropriate fitting parameters that are consistent with the scattering from components isolated via enzymatic digestions.

We estimate the relative crystalline cellulose content from the ($110/1\overline{1}0$) χ pole figures in Supplementary Fig. S6 for both dry and hydrated samples, given by $\int_{0}^{\pi /2} {\sin } \left( \chi \right){\text{I}}\left( \chi \right){\text{d}}\chi$, where χ is the azimuthal angle^23,52^. There is no statistically significant change in the relative crystalline cellulose content after drying. The values of the FWHM of the χ pole figures are 38.3 ± 1.8° and 40.0 ± 3.2° for hydrated and dried samples, respectively, with no statistically significant difference. Thus, drying does not perturb the degree of preferred crystal orientation with respect to the cell wall plane^24^.

TGA revealed 90.9 ± 1.7 and 7.0 ± 0.8 wt% water content for hydrated and air-dried onion epidermal peels, respectively (p < 0.001, Supplementary Fig. S7). The small amount of water in the dried samples is attributed to water that is bound to cellulose crystals^53^. Complete dehydration of the sample is likely not possible without compromising the integrity of the cell wall. Previous reports of TGA of oven-dried cellulose suggest irreversible changes after the removal of residual bound water^45^.

## Discussion

DFT models were used to predict how diffraction from cellulose would change in the presence of water while including the possibility of internal water molecules. Models 1 and 2 simulate a dry and hydrated CMF with no internal water molecules. In this case, the ($110/1\overline{1}0$) and (200) lattice spacings contract upon dehydration. Models 3 and 4 simulate a dry and hydrated CMF with water present in between cellulose sheets. When water is present within the crystal, we see no changes in the (200) lattice between dry and hydrated conditions, and only subtle changes in the ($110/1\overline{1}0$) reflections. These models, however, only capture a single CMF segment that is 4 glucan units long, whereas the actual primary cell wall is the average of many CMFs as well as other cell wall components.

The grazing-incidence geometry is crucial to enable X-ray scattering studies of hydrated primary cell walls because it allows for maximization of the signal-to-noise ratio of diffraction peaks while using a humidity chamber. The relative crystalline cellulose content or degree of preferred crystal orientation (texturing), calculated from the ($110/1\overline{1}0$) χ pole figure, did not change after drying. GIWAXS results indicate that the ($110/1\overline{1}0$) lattice spacing decreases by an average of 0.05 Å. This is observed in DFT Models 1 and 2, which do not have internal water. Conversely, the (200) reflection in the GIWAXS data did not change position after dehydration. This is observed in DFT Models 3 and 4, which had internal water molecules. Secondary cell wall has been reported to have the opposite effect, where the (200) spacing is larger and the (200) coherence length is smaller in the dried state^45,54,55^. This discrepancy may be due to differences in environments around cellulose in primary and secondary cell wall and in how the cell wall components interact with water. The experimental value of the (200) lattice spacing (Table 1) is also most consistent with Models 3 and 4, at 4.0 Å (Supplementary Table S1).

Neither set of DFT models fully captures the experimental results, but they still provide qualitative justification for the changes observed. The models have only a small, arbitrary amount of water molecules due to computational limitations. Therefore, we can expect that changes due to water loss can be more drastic in primary cell wall, which is > 90 wt% water, according to TGA measurements. We speculate that the different effects of dehydration on the ($110/1\overline{1}0$) and (200) lattice spacings are due to differences in hydrophilicity of the faces of the CMF. Hydroxymethyl groups on (110) and ($1\overline{1}0$) surfaces lead to more hydrophilicity than on (100) surfaces^56^. Water likely interacts more strongly with (110) and ($1\overline{1}0$) surfaces and may influence the crystalline structure.

The hydrogen-bonding network that binds glucan chains into sheets is integral to the crystalline ordering of cellulose Iβ^31,57^. Perturbations to this network would consequentially alter the crystalline structure of cellulose. Water in non-freezing and freezing states is known to bind to crystalline cellulose^53,58,59^. Inelastic neutron scattering spectroscopy studies of hydrated cellulose I suggested that bound non-freezing water interacts with outward facing hydroxymethyl groups, and that the hydrogen bonds within cellulose chains loosen when less water is present^59^. Raman spectroscopy of never-dried wood cellulose detected evidence of hydrogen bonding of water with cellulose^19^. Molecular modeling shows evidence of water hydrogen-bonding at crystalline cellulose interfaces^60^. Furthermore, single molecule AFM has suggested that hydration supports intrachain hydrogen bonding in individual cellulose molecules^61^, which we speculate may lead to the stabilization of cellulose crystals. Therefore, the removal of water may weaken cellulose-water and cellulose-cellulose hydrogen bonds in a manner that decreases the ($110/1\overline{1}0$) lattice spacing.

The intensity of the ($110/1\overline{1}0$) reflection relative to (200) increases upon dehydration in GIWAXS data as well as for both sets of DFT models. WAXS simulations of CMFs suggest the ratio of intensities between crystal lattice peaks depends on the arrangement of chains in microfibrils. Simulated WAXS diffractograms of CMFs also show larger ($110/1\overline{1}0$) intensity relative to the (200) intensity when either random uncorrelated or correlated disorder is imposed on the model^5^. In addition, the relative ($110/1\overline{1}0$) intensity varies from WAXS simulations of 18-chain microfibrils with different chain conformations^20^. The 333333 conformation has the largest ($110/1\overline{1}0$) to (200) intensity ratio, but is less energetically favorable than the 234432 and 34443 models (numbers represent the number of glucan chains per layer along the 200 direction of the crystal). Thus, the increase in the I($110/1\overline{1}0$) /I(200) peak ratio after drying (Table 1) may be due to an increase in chain packing disorder. The same trend is seen in XRD data from moisture-controlled wood, where the relative intensity of the ($110/1\overline{1}0$) reflection is larger in dry samples when compared to moist samples^54^. Altogether, this suggests that water may play a role in stabilizing cellulose crystals.

Enzymatic treatments of onion cell wall reveals that there are two major broad, amorphous peaks in GIWAXS data: a peak from pectin at ~ 0.85 Å^−1^ and a cuticle peak at 1.41 Å^-1^. Peak deconvolution (Fig. 3) reveals that the weakly-ordered pectin feature (Amorphous 1) is present both before and after dehydration (7.4 and 7.1 Å for hydrated and dried, respectively). The major pectin species in onion epidermal cell wall is homogalacturonan, comprising of about 50% of the cell wall^62^. The pectin matrix has previously been observed to collapse after dehydration, causing the overall cell wall thickness to decrease by 39% with little changes to lateral dimensions (perpendicular to the cell wall thickness) of onion peels^16^. We postulate that Amorphous 1 corresponds to a weakly ordered aggregate of pectin chains that is characteristic of the hydrated, unextracted state. This feature is not diminished after dehydration and collapse. Thus, we propose that little, if any, water is contained in pectin aggregates and that the bulk of the water is instead around widely-spaced pectins. The hydrophilic groups on pectin chains may make this structure more sensitive to surrounding water, which may explain why it appears to be more prominent in the scattering from the hydrated sample. The structure of the pectin aggregates may be a result of physical crosslinking (ionic or interchain interactions). Approximately 7.1 Å spacings have been observed in water-extracted pectin from fig seeds^12^ and dry commercial pectin powder^9^. Our results show that pectin is very weakly ordered and do not suggest the formation of highly crystalline nanofilaments, as previously suggested for Arabidopsis pavement cells^63^. The dehydration and collapse of the pectin matrix may also alter the surrounding environment of CMFs and may be another contributor to perturbation of the ($110/1\overline{1}0$) lattice spacing and peak width. Additionally, internal strain in the cellulose crystal due to drying may also be possible, as was previously observed in molecular dynamics simulations^64^ and vibrational sum frequency generation spectroscopy of onion epidermal peel^16^. Strain may contribute to broadening of the ($110/1\overline{1}0$) reflection after drying.

**Figure 4:**
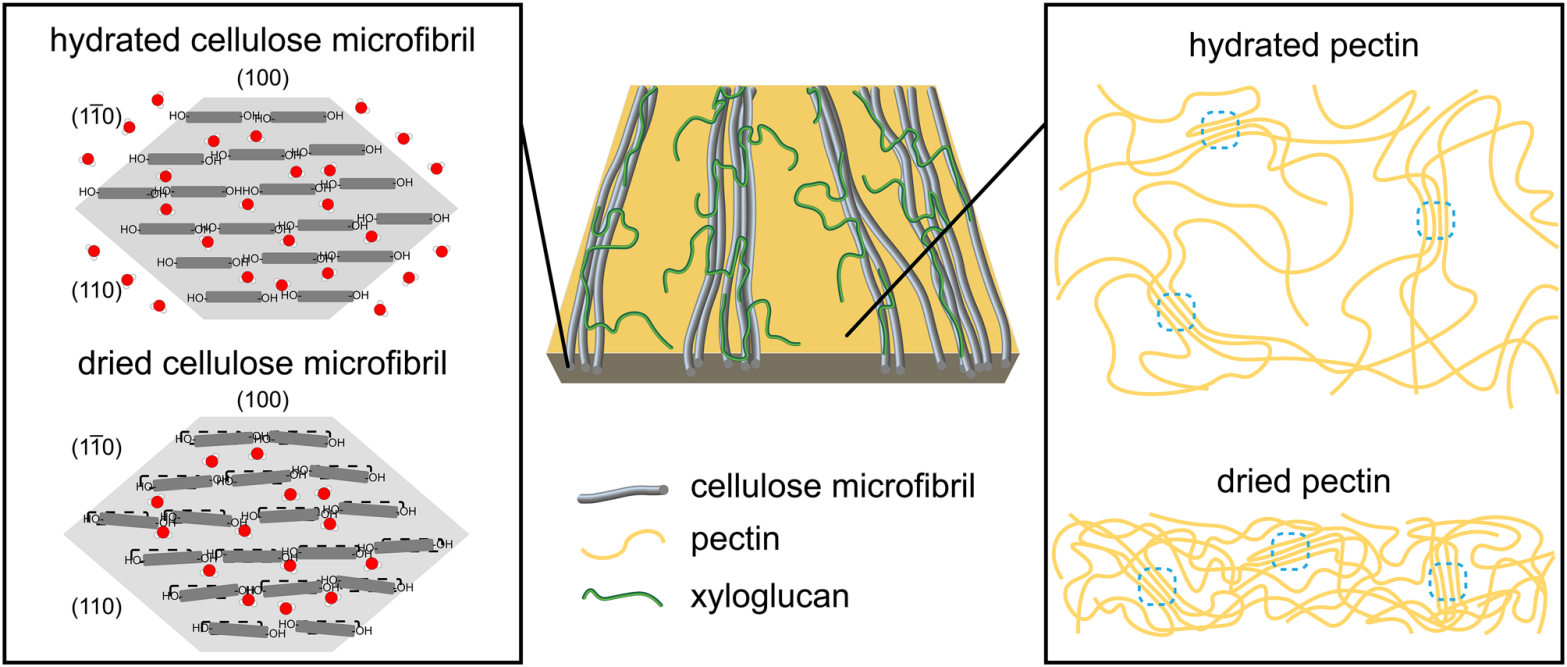
Proposed model of dehydration in primary cell wall. (Left) Changes in cellulose chain organization before and after dehydration. Dark gray bars indicate glucan chains. Water molecules outside of the light gray hexagon represent bulk water. Water interacts strongly with the (110) and ($1\overline{1}0$) surfaces in the hydrated state due to the presence of hydroxymethyl groups along the sides of the chains. Upon dehydration of bulk water, cellulose chains contract towards the center of the microfibril, parallel to (100), decreasing the ($110/1\overline{1}0$) spacing. The (200) lattice spacing does not collapse, possibly due to the presence of water molecules between the sheets. Chain packing disorder of glucan chains increases following dehydration. Dotted areas indicate the position of glucan rings prior to dehydration. (Middle) Illustration of a single lamella of primary cell wall based on coarse-grained simulations^65^. (Right) Changes in pectin assembly before and after dehydration. Water fills the gaps between loosely packed pectins, which vertically collapse after dehydration. A weakly ordered pectin chain aggregate, indicated by blue dashed lines, is preserved after the dehydration-induced collapse of the matrix.

We propose a model of the primary cell wall dehydration process. The left-hand side of Fig. 4 depicts an 18-chain model of a microfibril^5,66–68^, in a 234432 configuration^20^. When the cell wall is hydrated, bulk water outside of the microfibril is expected to have a stronger interaction with the ($110/1\overline{1}0$) surface than the (100) surface because of the hydroxymethyl groups on ($110/1\overline{1}0$). When the bulk water is removed, the hydrogen bonding network is disrupted such that the chains contract towards the middle of the microfibril, decreasing the average ($110/1\overline{1}0$) spacing by 0.05 Å. Because no change in the (200) spacing or coherence length is observed in GIWAXS experiments, chains must only shift parallel to the (100) plane. Trapped water molecules in between cellulose sheets may prevent the (200) spacing from collapsing. Removal of external water causes the chain positions to also become more disordered, illustrated in Fig. 4 as rotations of glucan chains. This corresponds to the relative intensity change of the ($110/1\overline{1}0$) peak with drying. In pectin, a 7.4 Å broad spacing is present in the hydrated state that corresponds to weakly ordered or aggregated pectin chains. When the cell wall dries, the matrix collapses mostly along the thickness of the cell wall, and the lateral spacing (in-plane with the cell wall) of the pectin chain aggregates persists and contracts by 0.3 Å (Fig. 4, right).

## Conclusion

We have used GIWAXS with a humidity chamber to enhance diffraction signal-to-noise ratios and minimize interference from scattering of water to examine the effect of dehydration on plant primary cell walls. Hydrated and dehydrated DFT models of CMFs and GIWAXS data of onions suggest that water stabilizes cellulose crystals. As such, dehydration perturbs the hydrogen bonding network and the crystal structure of cellulose in onion primary cell walls. GIWAXS also reveals an approximately 7 Å spacing that corresponds to the structure in native hydrated pectin that persists after dehydration despite the large collapse of the pectin matrix, due to this collapse occuringly mostly along the thickness of the cell wall.

## Supplementary Information

Supplementary Information.

## Data Availability

Raw and reduced data from this study are available from the corresponding authors upon reasonable request.
