# Supplementary material for: Grazing-incidence diffraction reveals cellulose and pectin organization in hydrated plant primary cell wall
Source: Sci Rep. 2023 Apr 3;13:5421. doi: 10.1038/s41598-023-32505-8 (PMC10070456; doi:10.1038/s41598-023-32505-8)
Supplement: Supplementary file 1 — Supplementary Information. [file 41598_2023_32505_MOESM1_ESM.pdf]

Supplementary Information for

Grazing-incidence diffraction reveals cellulose and pectin organization in hydrated plant primary cell wall

Joshua T. Del Mundo[1], Sintu Rongpipi[1], Hui Yang[2], Dan Ye[1], Sarah N. Kiemle[2], Stephanie L. Moffitt[3], Charles L. Troxel[3], Michael F. Toney[4], Chenhui Zhu[5], James D. Kubicki[6], Daniel J. Cosgrove[2], Esther W. Gomez[1,7], and Enrique D. Gomez[1,8,9*]*

[1]Department of Chemical Engineering, The Pennsylvania State University, University Park, PA 16802, USA

[2]Department of Biology, The Pennsylvania State University, University Park, PA 16802, USA

[3]SLAC National Accelerator Laboratory, Menlo Park, CA 94025, USA

[4]Department of Chemical and Biological Engineering and the Renewable and Sustainable Energy Institute, University of Colorado Boulder, Boulder, CO 80309, USA

[5]Advanced Light Source, Lawrence Berkeley National Laboratory, 1 Cyclotron Road, Berkeley, CA 94720, USA

[6]Department of Earth, Environmental and Resource Sciences, University of Texas at El Paso, El Paso, TX 79968, USA

[7]Department of Biomedical Engineering, The Pennsylvania State University, University Park, PA 16802, USA

[8]Department of Materials Science and Engineering, The Pennsylvania State University, University Park, PA 16802, USA

[9]Materials Research Institute, The Pennsylvania State University, University Park, PA 16802, USA

*email: ewg10@psu.edu (E. W. Gomez), edg12@psu.edu (E. D. Gomez)

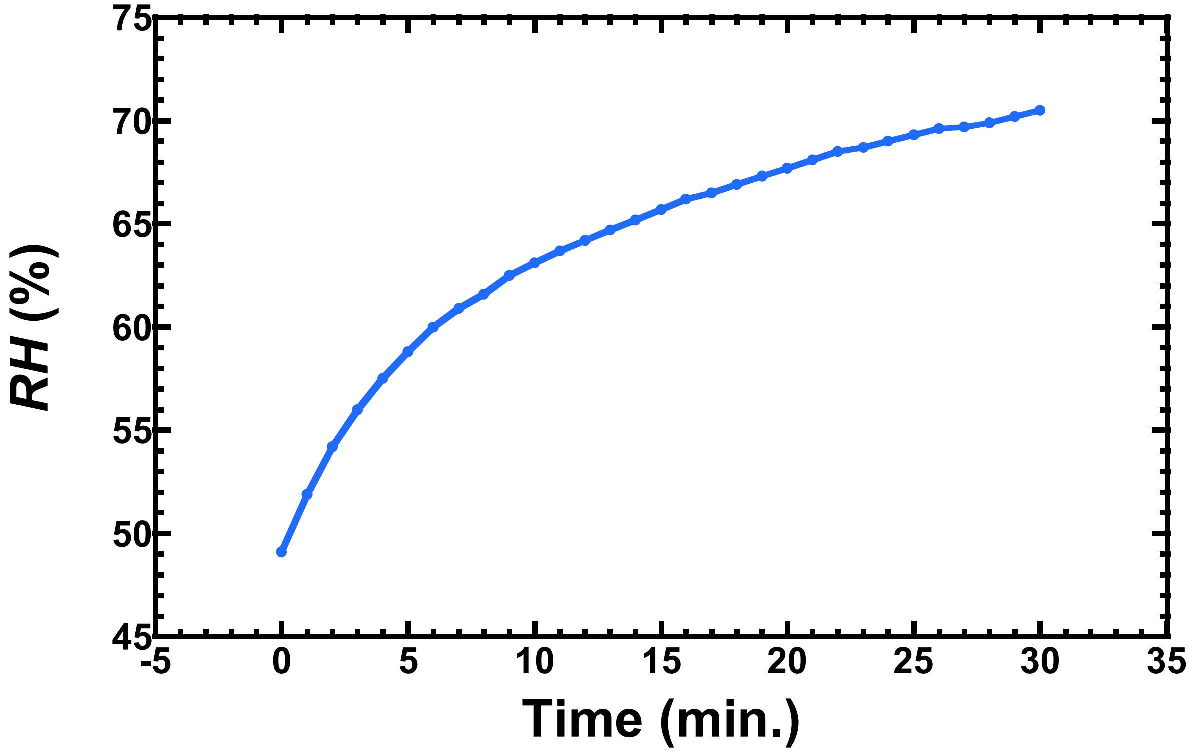

Figure S1. Relative humidity (RH) of GIWAXS sample chamber measured using a Govee wireless humidity sensor versus time after closing the chamber. All "wet" sample experiments took place after 20 min.

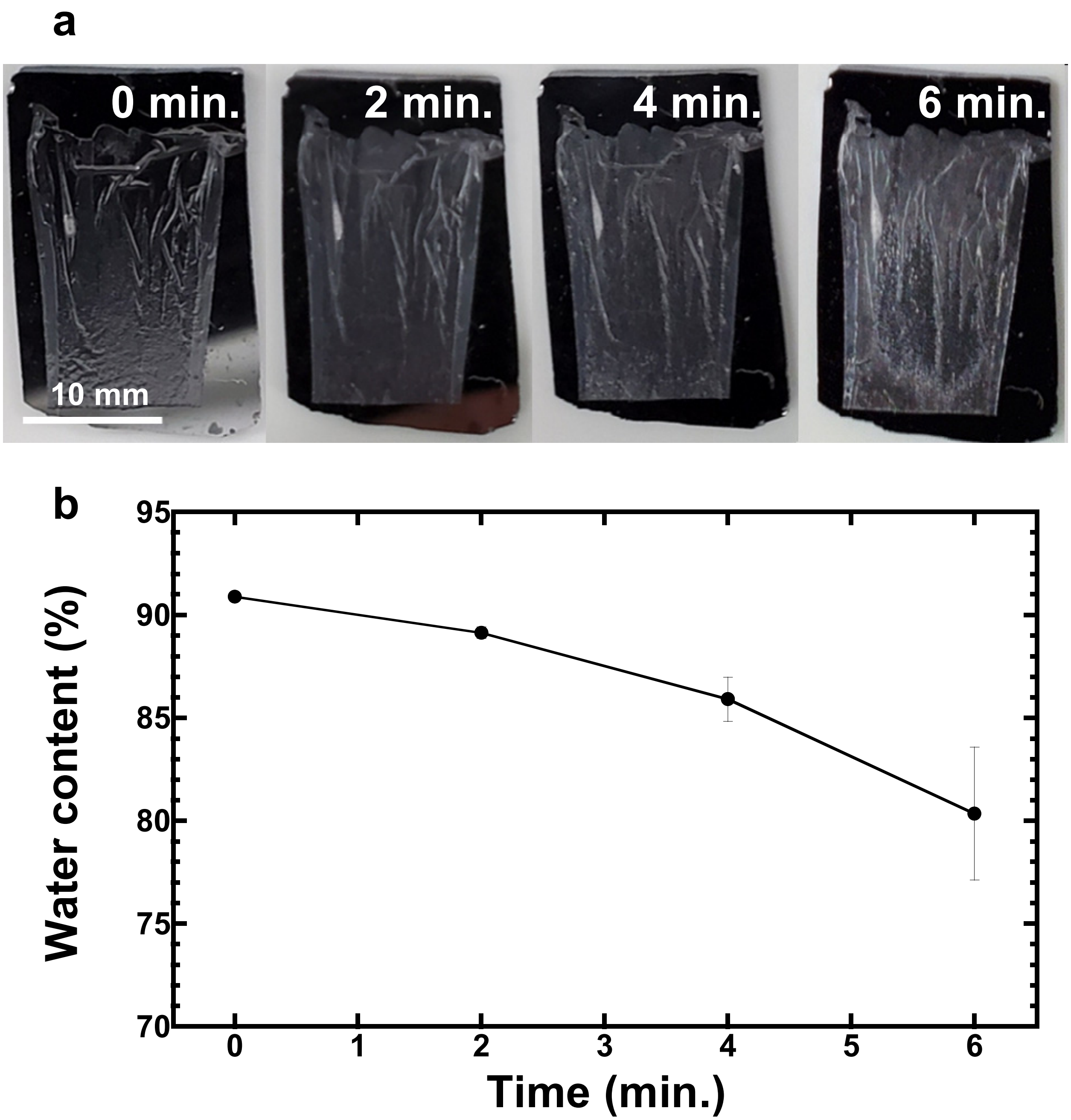

Figure S2. (a) Images of onion epidermal peel samples while drying in air (lab bench). Sample begins to show a dried appearance after 6 minutes of air-drying, starting from the edges. (b) Percent water content (water mass / total mass ×100) vs time of air-drying. Values shown are the averages over three samples. Error bars indicate standard deviation for multiple samples for each time point.

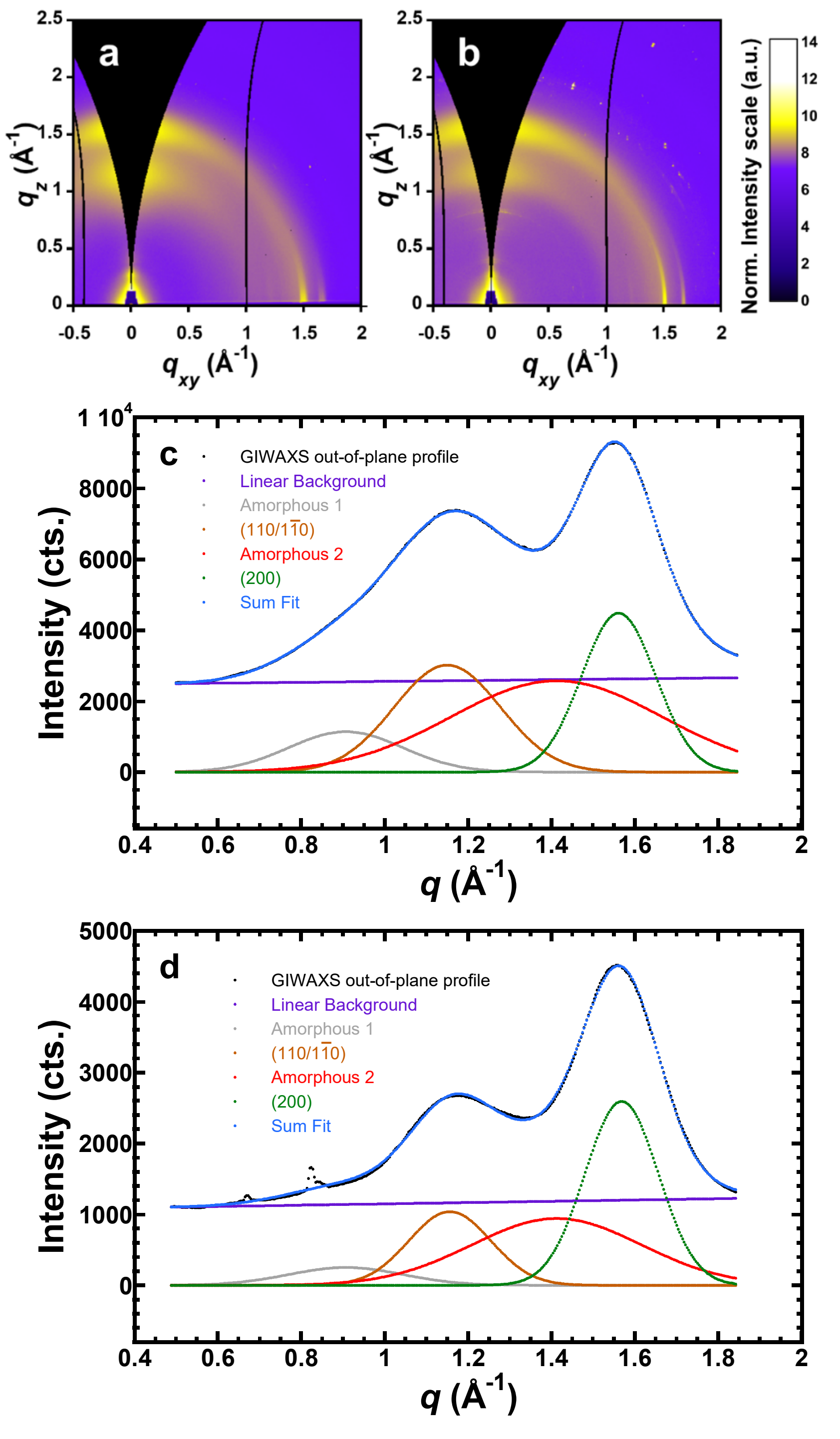

Figure S3. GIWAXS data of dried 5th scale onion epidermal wall. Data taken at the Advanced Light Source. (a) Untreated and (b) after pectate lyase digestion. Out-of-plane profiles and peak deconvolutions for (c) untreated and (d) after pectate lyase digestion. GIWAXS data are shown in black (GIWAXS out-of-plane profile), and components that make up a fit to the data (Sum Fit) are also shown. Amorphous 1 and Amorphous 2 correspond to broad peaks from matrix polysaccharides, amorphous cuticle components, and amorphous cellulose. The (110/1$\bar{1}$0) and (200) reflections are from crystalline cellulose for the Iβ allomorph.

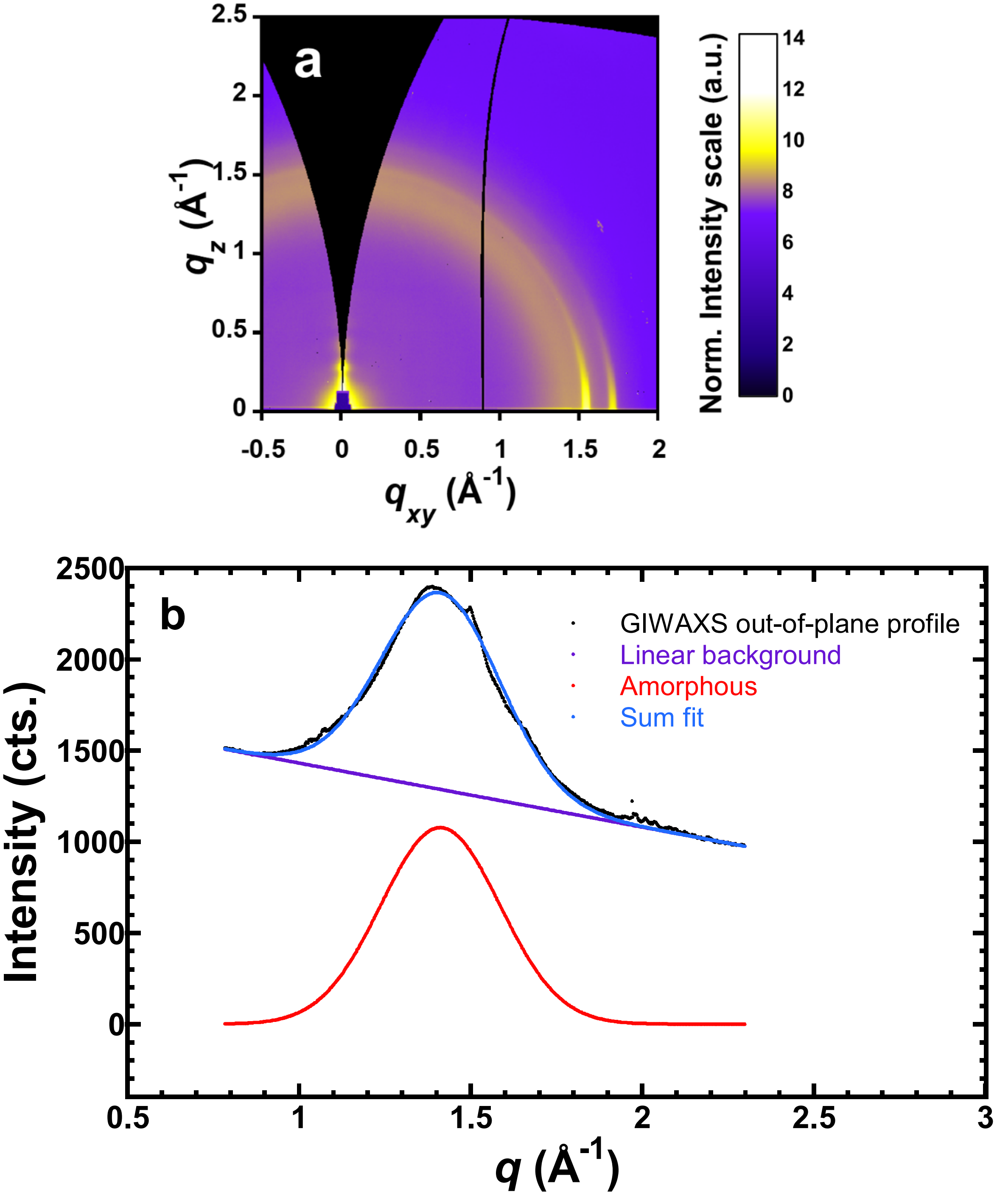

Figure S4. (a) GIWAXS data and (b) fitting of the out-of-plane GIWAXS profile from Driselase-treated 5th scale onion epidermal wall. Data taken at the Advanced Light Source. Components of the fit used to identify the peak position are shown.

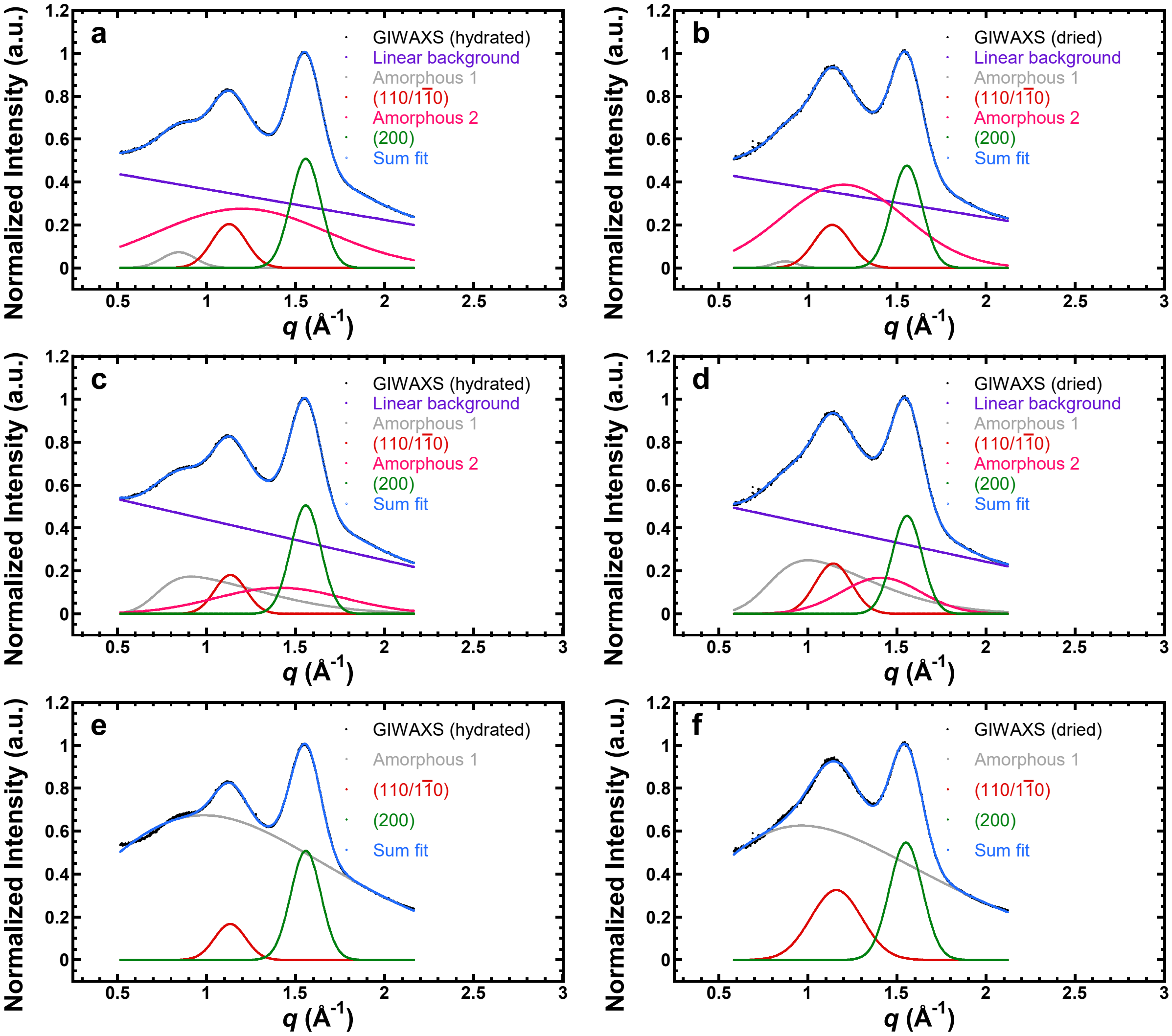

Figure S5. Examples of peak deconvolutions of the out-of-plane profiles from GIWAXS of (a,c,e) hydrated and (b,d,f) dried 5th scale onion epidermal wall using various alternative amorphous background fitting parameters: (a,b) Amorphous 2 fixed at q = 1.2 Å^{-1}, (c,d) Amorphous 1 modeled as an asymmetric Gaussian peak, and (e,f) a smooth asymmetric background.

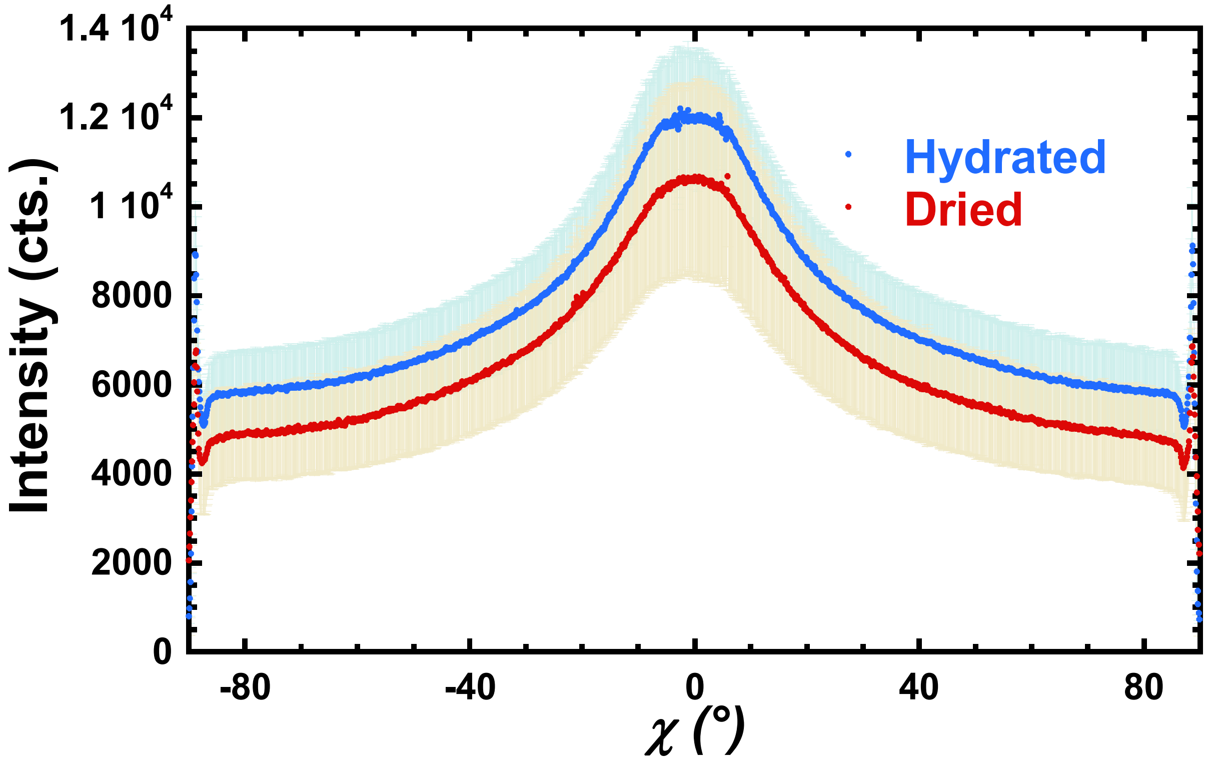

Figure S6. (110/1$\bar{1}$0) χ pole figures constructed from GIWAXS and rocking scans of hydrated and dried 5th scale onion epidermal wall. Data are averaged over three replicates. Error bars shown as light blue for hydrated onions and light orange for dried onions and represent the standard deviation over three measurements.

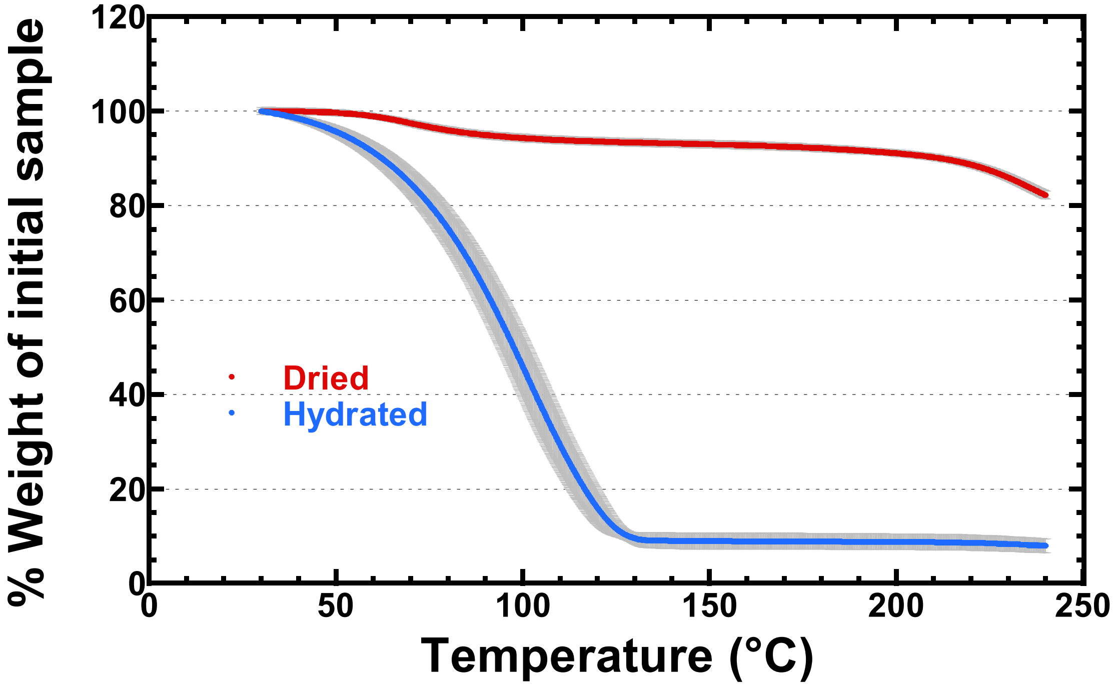

Figure S7. Thermogravimetric analysis of hydrated and dried 5th scale onion epidermal wall. Data are averaged over three replicates. Error bars represent standard deviation over three measurements.

Table S1. Peak positions in *q* and d-spacings of the (200) reflection from predicted scattering curves of DFT models with (1) no H_2O molecules, (2) monolayer of H_2O molecules on the outside of the cellulose crystal, (3) H_2O molecules inside the cellulose microfibril, and (4) H_2O molecules inside the cellulose microfibril plus a monolayer of H_2O molecules on the outside surface.

234432 model	q(200) ($Å^{-1}$)	d(200) (Å)
Model 1 (no water)	1.725	3.6
Model 2 (water monolayer)	1.650	3.8
Model 3 (water inside)	1.575	4.0
Model 4 (water in and out)	1.575	4.0

Table S2. Fit parameters q_{peak} (peak position), FWHM (full width at half maximum), and prefactor (intensity scaling factor outside the exponential) for gaussian fits to the peaks shown in Figure 3. Values shown are the sample average ± standard deviation, where n = 3. p-values were obtained using a paired student's t-test. *p<0.05, **p<0.01 in comparison of hydrated to dried samples.

		q_{peak} ($Å^{-1}$)	FWHM ($Å^{-1}$)	Prefactor
Amorphous 1	Hydrated	0.850 ± 0.002**	0.301 ± 0.010	0.32 ± 0.03
	Dried	0.890 ± 0.001	0.304 ± 0.014	0.41 ± 0.02
$(110/1\bar{1}0)$	Hydrated	1.128 ± 0.002**	0.240 ± 0.004*	0.53 ± 0.02**
	Dried	1.139 ± 0.002	0.277 ± 0.007	0.87 ± 0.02
Amorphous 2	Hydrated	1.413	0.770 ± 0.092*	0.41 ± 0.02**
	Dried	1.413	0.534 ± 0.013	0.64 ± 0.02
(200)	Hydrated	1.558 ± 0.003	0.198 ± 0.003	1.00
	Dried	1.562 ± 0.006	0.200 ± 0.003	1.00

Table S3. Ratio of d(110/1$\bar{1}$0) to d(200) and the coherence lengths of the (110/1$\bar{1}$0) and (200) reflections from the out-of-plane GIWAXS profiles of hydrated and dried 5th scale onion epidermal wall. Values shown are the sample average ± standard deviation, where n = 3. p-values were obtained using a paired student's t-test. *p<0.05 in comparison of hydrated to dried samples.

	**d(110/1$\bar{1}$0) / d(200)**	**L(110/1$\bar{1}$0)* (Å)**	**L(200) (Å)**
Hydrated	1.3815 ± 0.0007*	24.0 ± 0.3	29.1 ± 0.4
Dried	1.3721 ± 0.0020	20.8 ± 0.5	28.8 ± 0.5

Table S4. Lattice spacings of the (110/1$\bar{1}$0) and (200) planes and the ratio of the peak intensity at (110/1$\bar{1}$0) to that of (200) reflections from the out-of-plane GIWAXS profiles of hydrated and dried 5th scale onion epidermal wall for alternative fitting parameters to those used in Figure 3 of the main text. Values shown are the sample average ± standard deviation, where n = 3. p-values obtained by comparing hydrated and dried using a paired student's t-test. *p<0.05, **p<0.01, and ***p<0.001 in comparison of hydrated to dried samples

		**d(110/1$\bar{1}$0) (Å)**	**d(200) (Å)**	**I(110/1$\bar{1}$0) / I(200)**
Amorphous 2 fixed at q = 1.2 $Å^{-1}$	Hydrated	5.57 ± 0.01*	4.03 ± 0.01	0.41 ± 0.02
	Dried	5.51 ± 0.02	4.02 ± 0.01	0.46 ± 0.03
Asymmetric Gaussian Amorphous 1	Hydrated	5.54 ± 0.01*	4.03 ± 0.01	0.36 ± 0.02**
	Dried	5.48 ± 0.01	4.02 ± 0.01	0.56 ± 0.03
Asymmetric amorphous background	Hydrated	5.54 ± 0.01***	4.03 ± 0.01	0.32 ± 0.02**
	Dried	5.40 ± 0.01	4.03 ± 0.01	0.62 ± 0.03
